# Predictive Value of the Triglyceride-Glucose Index (TyG Index) for Major Cardiac Events in Patients With Chronic Heart Failure and Type 2 Diabetes: A Prospective Observational Study

**DOI:** 10.7759/cureus.103232

**Published:** 2026-02-08

**Authors:** Md. Fakhrul Hasan, Manzoor Mahmood, Mohmmad D Selim, Abu Salim, Dipal Krishna Adhikary, Khurshid Ahmed

**Affiliations:** 1 Department of Cardiology, National Institute of Cardiovascular Diseases, Dhaka, BGD; 2 Department of Cardiology, Bangladesh Medical University, Dhaka, BGD; 3 Department of Cardiology, Bangladesh Specialized Hospital, Dhaka, BGD

**Keywords:** heart failure, insulin resistance, major adverse cardiac events, prognosis, triglyceride glucose index, type 2 diabetes mellitus

## Abstract

Introduction: Chronic heart failure (CHF) with coexisting type 2 diabetes mellitus (T2DM) confers a markedly elevated risk of major adverse cardiac events (MACEs). Insulin resistance plays a central pathophysiological role in this excess risk, yet direct assessment remains impractical in routine care. The triglyceride-glucose (TyG) index, a straightforward surrogate indicator of insulin resistance derived from fasting glucose and triglyceride levels, has emerged as a potential prognostic tool. This study evaluated the predictive value of the TyG index for MACE in patients with CHF and T2DM.

Method: This prospective observational study was conducted at a tertiary care cardiology center in Bangladesh from July 2023 to August 2024. Eighty-three adults with confirmed CHF and T2DM were enrolled and followed for six months. Baseline clinical, echocardiographic, and biochemical parameters were recorded, and the TyG index was calculated using a validated formula. Major adverse cardiac events, including cardiovascular death, heart-failure-related hospitalization, and ischemic cardiac events, were adjudicated by blinded cardiologists. Group comparisons, quartile analysis, and receiver operating characteristic (ROC) curve analysis were performed to evaluate the prognostic performance.

Results: Major adverse cardiac events occurred in 69 (83.1%) of patients, predominantly due to heart-failure-related hospitalization in 67 (80.7%). The TyG index was significantly higher in patients with MACE compared with those without MACE (6.8 ± 0.7 vs. 5.8 ± 0.6; p=0.001). Higher TyG quartiles were significantly associated with mortality and heart-failure hospitalization (p<0.05). ROC analysis demonstrated excellent predictive accuracy for MACE (AUC=0.890; 95% CI: 0.818-0.962), with an optimal cutoff of ≥6.30 yielding 53/69 (76.8%) sensitivity and 13/14 (92.9%) specificity.

Conclusion: The TyG index is a strong and clinically useful predictor of major adverse cardiac events in patients with coexisting CHF and T2DM. Its simplicity and accessibility support its integration into routine risk stratification, particularly in resource-limited settings.

## Introduction

Chronic heart failure (CHF) and type 2 diabetes mellitus (T2DM) represent one of the most common chronic non-communicable diseases globally and together account for a substantial proportion of global cardiovascular morbidity and mortality [1]. The concurrent rise in the prevalence of both conditions has intensified the clinical challenge of identifying patients at heightened risk of adverse outcomes, underscoring the importance of reliable risk stratification strategies to improve prognosis and guide management [1-4]. Individuals with T2DM who also suffer from CHF constitute a particularly vulnerable subgroup, as the coexistence of these conditions markedly increases the likelihood of major adverse cardiac events (MACEs). Early identification of residual metabolic and cardiovascular risk factors in this population is therefore essential to optimize clinical decision-making and reduce future cardiovascular events [1-3].

Insulin resistance (IR) represents a central pathophysiological abnormality in T2DM and plays a critical role in the initiation and progression of cardiovascular disease, including CHF [1-3]. Through multiple interrelated mechanisms, such as endothelial dysfunction, dyslipidemia, chronic inflammation, and the promotion of hypertension, IR contributes directly to the development of adverse cardiovascular outcomes and MACE [1]. Although the hyperinsulinemic-euglycemic clamp remains the reference standard for the assessment of IR, its technical complexity, high cost, and limited feasibility restrict its use in routine clinical practice and large-scale epidemiological studies [1]. Owing to the close relationship between IR and persistently elevated levels of plasma glucose and triglycerides, attention has shifted toward simple surrogate indices derived from commonly available laboratory parameters [1]. Among these, the triglyceride-glucose (TyG) index calculated using fasting plasma glucose and triglyceride concentrations has emerged as a practical, reproducible, and validated marker of IR [1]. Several investigations have demonstrated a strong correlation between the TyG index and IR measured by both the hyperinsulinemic-euglycemic clamp and the Homeostasis Model Assessment of Insulin Resistance (HOMA-IR) [1].

Beyond its role as a surrogate marker of IR, the TyG index has attracted increasing interest for its prognostic implications in cardiovascular disease. Elevated TyG index values have been repeatedly linked to a higher incidence of cardiovascular events and unfavorable cardiovascular outcomes across diverse clinical settings [3-5]. In particular, the TyG index has been shown to predict MACE among patients with coronary artery disease (CAD), including those undergoing percutaneous coronary intervention (PCI) in the context of acute coronary syndrome [6,7]. Associations between higher TyG levels and increased cardiovascular risk have also been reported in other high-risk populations, such as individuals with hypertension, chronic kidney disease, hyperuricemia, and atrial fibrillation [1].

Growing evidence further supports the relevance of the TyG index in the broader spectrum of cardiovascular disease progression. Higher TyG index values have been associated with accelerated atherosclerosis, increased rates of ischemic events, and overall worsening of cardiovascular status [6-8]. Meta-analyses and longitudinal cohort studies have consistently demonstrated its predictive value for MACE in patients with CAD and those treated with PCI [6,7]. Importantly, emerging data suggest that the TyG index may also have prognostic significance in heart failure, showing associations with left ventricular global longitudinal strain--a sensitive indicator of subclinical myocardial dysfunction--as well as with long-term mortality and cardiovascular events in patients with CHF [3,9,10]. Despite these advances, evidence specifically addressing the long-term prognostic value of the TyG index for MACE in patients with the combined burden of CHF and T2DM remains limited. This gap is particularly relevant in low- and middle-income countries, where the prevalence of these conditions is high and access to advanced diagnostic modalities is often constrained [2]. The scarcity of prospective observational studies in this specific population has hindered the incorporation of the TyG index into regional risk stratification frameworks. Accordingly, the present study was designed to evaluate the predictive significance of the TyG index for major adverse cardiovascular events in patients with coexisting CHF and T2DM.

## Materials and methods

Study place and time

This prospective observational study was carried out in the Department of Cardiology of a tertiary-level hospital in Bangladesh over a 14-month period, from July 15, 2023, to August 31, 2024. The study was conducted in accordance with the principles of the Declaration of Helsinki and received approval from the institutional review board prior to the study initiation (approval number: BSMMMU/2023/9310). Each participant gave written informed consent before study enrollment.

Study population

A total of 83 adult patients aged 18 years or older were consecutively recruited from admissions to the Department of Cardiology. Eligibility for inclusion required a confirmed diagnosis of chronic heart failure, established on the basis of compatible clinical features in combination with elevated natriuretic peptide concentrations, defined as brain natriuretic peptide levels above 35 pg/mL or N-terminal pro-brain natriuretic peptide levels above 125 pg/mL, together with echocardiographic evidence of structural cardiac abnormalities [11]. In addition, all enrolled patients were required to have a diagnosis of T2DM confirmed according to the American Diabetes Association diagnostic criteria [12]. Patients were excluded if they had prediabetes or type 1 diabetes mellitus, evidence of active malignancy, sepsis, severe anemia with a hemoglobin level below 6 g/dL, advanced chronic kidney disease characterized by an estimated glomerular filtration rate (eGFR) less than 30 mL/min/1.73 m², severe hepatic impairment, or if they were receiving hormone therapy at the time of enrollment.

Sample size calculation

The sample size was calculated using the following formula:

$n = \frac{\left\{ Z_{\alpha}\sqrt{\pi(1-\pi)} + Z_{\beta}\sqrt{\pi_0(1-\pi_0)} \right\}^2}{(\pi - \pi_0)^2}$.

In this equation, n denotes the minimum required sample size. Zα represents the standard normal deviate corresponding to a two-sided significance level of 5% and was set at 1.96, while Zβ denotes the standard normal deviate corresponding to a statistical power of 80% and was set at 0.84. π0 indicates the expected proportion of MACE among patients with chronic heart failure and T2DM based on previously published data and was taken as 37.8% (π0=0.378) [13]. π represents the projected proportion of MACE assumed for the present study population and was set at 25.0% (π=0.25). Using these parameters, the calculated minimum sample size was 72 participants. To account for potential loss to follow-up, after allowing for an anticipated 10% attrition rate, the final planned sample size was set at 80 participants.

Data collection

Baseline information was obtained systematically using a pre-validated, semi-structured data collection instrument. Recorded variables included demographic characteristics, relevant clinical comorbidities such as hypertension and dyslipidemia, and physiological parameters including blood pressure and heart rate. Left ventricular ejection fraction (LVEF) was evaluated by transthoracic echocardiography. Venous blood samples were obtained after an overnight fasting period of at least 12 hours. The obtained blood samples were processed to measure fasting plasma glucose and a comprehensive lipid profile, including total cholesterol, high-density lipoprotein (HDL) cholesterol, low-density lipoprotein (LDL) cholesterol, and triglycerides, as well as glycated hemoglobin (HbA1c), serum creatinine, and inflammatory biomarkers. The triglyceride-glucose (TyG) index was subsequently derived using the following validated equation [14,15]:

$\text{TyG index} = \ln \left[ \frac{\text{fasting triglyceride (mg/dL)} \times \text{fasting glucose (mg/dL)}}{2} \right]$.

Follow-up and outcome ascertainment

Following hospital admission, participants were monitored prospectively for a duration of six months to document the occurrence of major adverse cardiovascular events (MACEs). The primary outcome was defined as a composite of cardiovascular mortality, hospitalization attributable to worsening heart failure, and ischemic cardiac events, including myocardial infarction and unstable angina requiring coronary revascularization [16]. Based on the presence or absence of any of these outcomes during the follow-up period, patients were classified into either the MACE group or the non-MACE group. Event adjudication was independently performed by two cardiologists who were blinded to the TyG index values.

Statistical analysis

Statistical analyses were performed using IBM Statistical Package for the Social Sciences (SPSS), version 26 (IBM Corp., Armonk, New York, USA). The Shapiro-Wilk test was performed to assess the normality of data. Variables following a normal distribution were summarized as mean with standard deviation, while those showing skewed distributions were described using median values and ranges. Differences in continuous variables between participants with and without major adverse cardiovascular events were examined using the independent-samples t-test for normally distributed data and the Mann-Whitney U test for non-normally distributed data. Categorical data were expressed as frequencies with proportions presented in percentage form, and the chi-square test or Fisher’s exact test was employed (when expected frequencies were fewer than five) for evaluating group comparisons. The discriminative performance of the triglyceride-glucose (TyG) index for predicting major adverse cardiovascular events was assessed through receiver operating characteristic (ROC) curve analysis, with the optimal cutoff value identified by the maximum Youden index. A two-tailed P-value of less than 0.05 was considered statistically significant.

## Results

Demographic profile

Among the total participants, 3 (3.6%) were aged 18-38 years, 29 (34.9%) were aged 39-58 years, 42 (50.6%) were aged 59-78 years, and 9 (10.8%) were over 78 years old. The mean age was 60.64 ± 13.4 years, with a median age of 62 years, spanning a range of 72 years. In terms of sex distribution, 58 (69.9%) participants were male, whereas 25 (30.1%) were female (Table 1).

**Table 1:** Demographic profile of the study participants (n=83). Data are expressed as frequencies (percentages) and mean ± SD.

Demographic profile	Category	Frequency (%)
Age (years)	18-38	3 (3.6%)
	39-58	29 (34.9%)
	59-78	42 (50.6%)
	>78	9 (10.8%)
	Mean ± SD	60.64 ± 13.4
Sex	Male	58 (69.9%)
	Female	25 (30.1%)

Major adverse cardiac event (MACE) incidence and clinical outcome

Approximately 83.10% of the respondents had major cardiac events, whereas 16.90% had no major cardiac events. Among the patients who had MACE as an outcome, 7 (8.43%) patients died, 9 (10.84%) patients experienced ischemic cardiac events, and 67 (80.72%) were hospitalized due to HF (Table 2).

Approximately 83.10% of the respondents had major cardiac events, whereas 16.90% had no major cardiac events. Among the patients who had MACE as an outcome, 7 (8.43%) patients died, 9 (10.84%) patients experienced ischemic cardiac events, and 67 (80.72%) patients were hospitalized due to HF (Table 2).

**Table 2:** Distribution of the participants according to MACEs and outcomes (n=83). Data are presented as frequencies (percentages). MACE: major adverse cardiac event, HF: heart failure.

MACE	Frequency (%)
Yes	69 (83.1%)
No	14 (16.9%)
Clinical outcome	
Death	7 (8.43%)
Ischemic cardiac events	9 (10.84%)
Hospitalization due to HF	67 (80.72%)

Associations between the triglyceride-glucose (TyG) index and major adverse cardiac events (MACEs)

**Figure 1:**
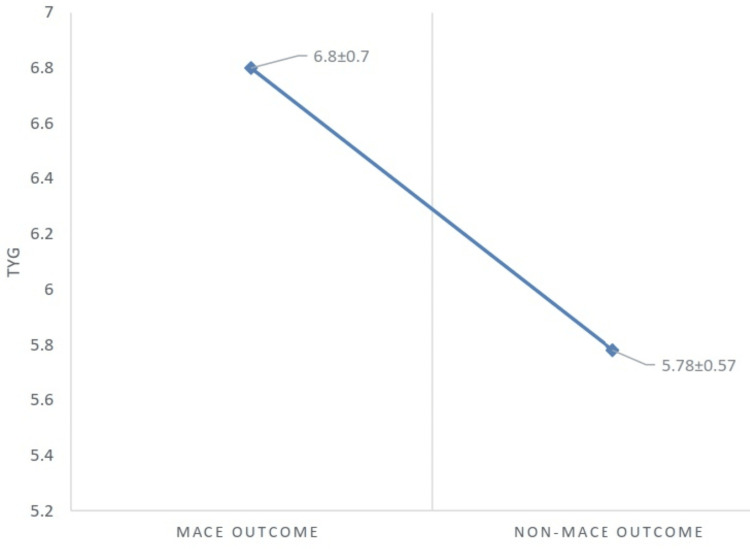
Distribution of the TyG index among MACE and non-MACE outcomes. MACE: major adverse cardiac events, TyG: triglyceride-glucose.

Participants who experienced major adverse cardiovascular events exhibited significantly higher triglyceride-glucose (TyG) index values compared with those who did not develop MACE (6.8 ± 0.7 vs. 5.8 ± 0.57). This difference demonstrates a statistically significant relationship between elevated TyG index levels and the occurrence of major adverse cardiovascular events (p=0.001), as illustrated in Figure 1.

Associations between triglyceride-glucose (TyG) and clinical outcomes

Of the seven deaths, 5 (71.4%) occurred in patients in higher quartiles. Q3 and Q4 of TyG were also significantly associated with hospitalization due to HF (Table 3).

**Table 3:** Outcomes in different TyG tertiles. *p<0.05; **p<0.001. TyG: triglyceride-glucose, HF: heart failure.

Outcomes	TyG index				P-value
	Q1 (21)	Q2 (20)	Q3 (21)	Q4 (21)	
Death	2 (28.6%)	0	5 (71.4%)	0	0.017*
Ischemic events	2 (22.2%)	1 (11.1%)	3 (33.3%)	3 (33.3%)	0.739
Hospitalization due to HF	11 (16.7%)	15 (22.7%)	21 (31%)	20 (29%)	<0.001**

Predictive value of the triglyceride-glucose (TyG) index

**Figure 2:**
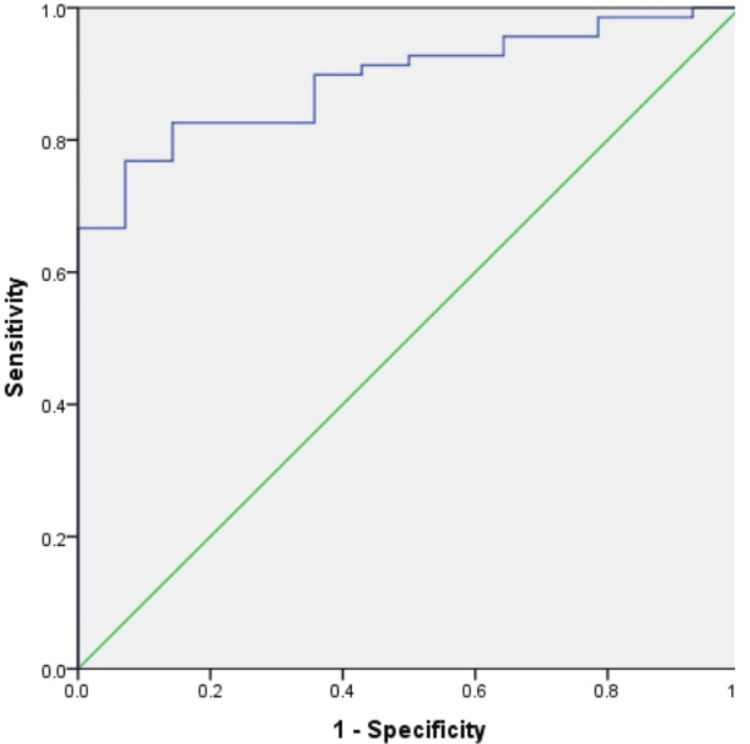
ROC analysis. ROC: receiver operating characteristic.

Receiver operating characteristic (ROC) curve analysis demonstrated that the triglyceride-glucose (TyG) index had strong discriminative ability for predicting adverse outcomes. The area under the curve (AUC) was 0.890 (95% CI: 0.818-0.962) (Figure 2). A TyG index cutoff of ≥6.30 yielded the highest Youden index (0.697) with a sensitivity of 53/69 (76.8%) and a specificity of 13/14 (92.9%) (Table 4).

**Table 4:** Determination of the AUC, cutoff value, sensitivity, and specificity of TyG. AUC: area under the curve, TyG: triglyceride-glucose, NPV: negative predictive value, PPV: positive predictive value.

AUC	Cutoff value	Sensitivity	Specificity	PPV	NPV	Accuracy
0.890	6.30	53/69 (0.768)	13/14 (0.929)	53/54 (0.981)	13/29 (0.448)	66/83 (0.795)

## Discussion

The current study aimed to evaluate the prognostic significance of the triglyceride-glucose (TyG) index for major adverse cardiovascular events (MACE) in patients with concomitant chronic heart failure (CHF) and type 2 diabetes mellitus (T2DM). The findings demonstrate a significant relationship between higher TyG index levels and an increased risk of MACE, with heart-failure-related hospitalizations accounting for the majority of observed events. These results highlight the important contribution of insulin resistance, as captured by the TyG index, to adverse clinical outcomes in this high-risk population, supporting its clinical value as a practical and easily obtainable biomarker [1-4].

The study population comprised predominantly older adults with a male predominance, consistent with the established epidemiological profiles of both CHF and T2DM. Older age is a well-recognized risk factor for the development and progression of both conditions, while cardiovascular diseases generally exhibit a higher incidence and prevalence in men [1,2]. These demographic characteristics are congruent with findings from previous large-scale heart failure and cardiometabolic studies, which consistently report an increasing burden of these diseases in an aging global population [2,9].

A notable finding was the high overall incidence of MACE, primarily attributed to heart-failure-related hospitalizations. This observation highlights the advanced stage of CHF in the study cohort and the substantial contribution of diabetes to adverse outcomes. Patients with coexisting CHF and T2DM represent a particularly high-risk subgroup, where T2DM exacerbates myocardial dysfunction, promotes maladaptive remodeling, and accelerates the progression of heart failure, leading to frequent decompensation events requiring hospitalization [2,9,10].

The significantly higher TyG index observed in patients who experienced MACE provides compelling evidence for its prognostic value. The TyG index serves as a reliable surrogate marker for insulin resistance (IR), a key pathophysiological mechanism linking T2DM to adverse cardiovascular outcomes [1-3,9,17]. Elevated IR contributes to myocardial energy dysregulation, shifting substrate utilization from glucose to fatty acids, which impairs cardiac efficiency and promotes lipotoxicity [9]. Furthermore, IR is intricately involved in endothelial dysfunction, fostering a pro-inflammatory state and oxidative stress that damage the vascular endothelium, leading to macro- and microvascular complications crucial in both CHF and T2DM progression [6]. It also contributes to ventricular remodeling through fibrosis and hypertrophy, ultimately worsening cardiac function [9].

A growing body of evidence from prior investigations supports the association between elevated triglyceride-glucose (TyG) index values and unfavorable cardiovascular outcomes across multiple patient populations. Previous studies have shown that higher TyG levels are linked to an increased incidence of major adverse cardiovascular events among individuals with chronic coronary syndrome undergoing percutaneous coronary intervention, as well as among patients with coronary artery disease and coexisting hypertension [17,18]. Comparable prognostic relationships have also been observed for long-term mortality and MACE in patients with type 2 diabetes mellitus, in addition to an elevated risk of worsening heart failure and all-cause mortality among older patients with chronic heart failure and T2DM [2,4]. Taken together, these consistent findings across a wide spectrum of cardiovascular conditions underscore the broader applicability of the TyG index as a prognostic marker.

The observed dose-response relationship, where higher TyG quartiles (Q3 and Q4) were strongly associated with mortality and heart failure hospitalization, further strengthens the prognostic significance of the TyG index. This gradient in risk suggests that increasing levels of IR, as indicated by higher TyG values, correspond to a progressively worse cardiovascular prognosis. This pattern is consistent with the understanding that IR is a continuous metabolic derangement, where higher degrees of IR lead to more profound and widespread pathophysiological consequences on cardiovascular health [18].

The excellent discriminatory ability of the triglyceride-glucose (TyG) index, reflected by a high area under the receiver operating characteristic curve and an optimal cutoff threshold, emphasizes its practical clinical value. As an easily accessible and inexpensive marker for risk stratification, the TyG index offers clear advantages over more technically demanding and costly methods used to assess insulin resistance, including the HOMA-IR [1-3,9]. This advantage is particularly important in low- and middle-income settings, such as Bangladesh, where advanced metabolic testing and specialized imaging facilities are often not readily available. The use of routine laboratory parameters to identify individuals at increased cardiovascular risk has the capacity to support improved clinical decision-making and optimizing the allocation of healthcare resources in these environments.

This study has several limitations. The generalizability of the results may be constrained by the single-center nature of the study and the small sample size, although the consistency of the results with those reported in larger international studies partly mitigates this concern. Despite adjustment for relevant confounding variables, the possibility of residual confounding due to unmeasured factors cannot be entirely excluded. Further multicenter investigations involving larger cohorts and extended follow-up periods are needed to confirm these findings and to further evaluate the predictive value of the TyG index across different clinical subgroups.

## Conclusions

The findings of this study indicate that the triglyceride-glucose (TyG) index has significant prognostic value for MACE, particularly hospitalizations related to heart failure, in patients with coexisting CHF and T2DM. As a simple and readily available biomarker, the TyG index may facilitate early identification of individuals at increased risk, allowing for closer surveillance and more intensive management. Further research is required to determine the role of the TyG index within routine cardiovascular risk assessment frameworks and to assess its impact on clinical outcomes through prospective interventional studies.
